# Prevalence of Antimicrobial Resistance in *Klebsiella pneumoniae* in the South African Populations: A Systematic Review and Meta‐Analysis of Surveillance Studies

**DOI:** 10.1002/mbo3.70037

**Published:** 2025-07-24

**Authors:** Sinethemba H. Yakobi, Uchechukwu U. Nwodo

**Affiliations:** ^1^ Patho‐Biocatalysis Group (PBG), Department of Biochemistry and Biological Sciences University of Fort Hare Alice South Africa

**Keywords:** antimicrobial resistance, *Klebsiella Pneumoniae*, meta‐analysis, public health, systematic review

## Abstract

Antimicrobial resistance (AMR) poses a critical global health threat, with *Klebsiella pneumoniae* emerging as a high‐priority pathogen due to escalating resistance rates. This systematic review and meta‐analysis evaluated the AMR profiles of *K. pneumoniae* isolates from South Africa, a resource‐limited setting where AMR burdens remain understudied. A comprehensive search of PubMed, Web of Science, and Google Scholar (January 2000 to June 2024) identified studies reporting resistance data. Nineteen studies comprising 9402 isolates were included, and data were analyzed using random‐effects models. Pooled resistance prevalence was highest for amoxicillin (69.3%; 95% CI: 64.1%–74.1%), followed by second‐generation cephalosporins (70.9%; 95% CI: 65.3%–75.8%), trimethoprim‐sulfamethoxazole (57.8%; 95% CI: 52.4%–63.0%), and carbapenems, with imipenem resistance at 33.2% (95% CI: 28.5%–38.3%). Significant heterogeneity was observed (*I²* > 90%, *p* < 0.001), likely due to differences in study populations such as clinical versus environmental isolates, and regional prescribing practices. Publication bias was detected (Egger's test: *p* = 1.45 × 10⁻¹⁴), indicating possible underreporting of small‐scale studies with null findings. These findings highlight alarming resistance rates to first‐line antibiotics in South Africa and underscore the urgent need for multisectoral interventions. Priority actions should include standardized AMR surveillance to harmonize data collection, expanded antimicrobial stewardship programs particularly in high‐resistance settings such as hospitals, and greater investment in novel therapies targeting carbapenem‐resistant strains. Addressing methodological heterogeneity and minimizing publication bias in future research will be critical to strengthening the evidence base for informed policymaking.

## Graphical abstract

*Klebsiella pneumoniae* isolates in South Africa exhibit > 50% resistance to first‐line antibiotics, with rising carbapenem resistance. Urgent, targeted interventions and genomic surveillance are essential to curb the spread of multidrug‐resistant strains and preserve treatment efficacy.

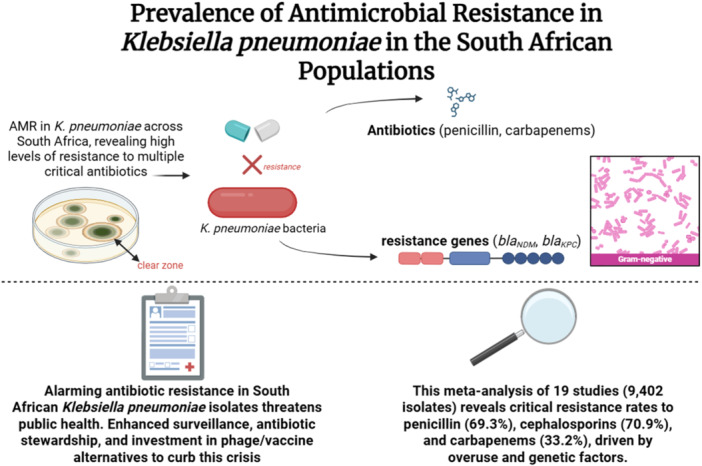

## 1 Introduction

Antimicrobial resistance (AMR) represents a growing global health emergency, recognized by the World Health Organization (WHO) as one of the top 10 public health threats facing humanity (Yakobi et al. 2024; Yakobi and Pooe 2022). The rapid rise of AMR undermines decades of medical progress, as once‐effective treatments become increasingly obsolete (Ahmed et al. 2024). Among the most concerning pathogens is *Klebsiella pneumoniae*, a gram‐negative opportunistic bacterium known for its role in hospital‐acquired infections and its remarkable ability to acquire resistance to multiple antibiotic classes (Abbas et al. 2024). *K. pneumoniae* causes a range of serious infections, including pneumonia, bloodstream infections, wound infections, and urinary tract infections, and is a key member of the ESKAPE group of MDR organisms (Asokan et al. 2025). While AMR is a global issue, its burden is particularly acute in low‐ and middle‐income countries (LMICs), where health systems face resource constraints and limited surveillance capacity (Shamas et al. 2023). In South Africa, the challenge is compounded by the intersection of AMR with high prevalence rates of HIV, tuberculosis, and noncommunicable diseases, all of which increase vulnerability to bacterial infections and drive antibiotic consumption (Modjadji 2021; Peer 2015). Recent studies in South Africa have documented alarming rates of resistance to first‐line antibiotics such as beta‐lactams and aminoglycosides in *K. pneumoniae* isolates, raising urgent concerns for public health (Finlayson et al. 2024; Kariuki et al. 2022; Osei Sekyere 2016). Despite these findings, there is a lack of consolidated national data on resistance patterns in *K. pneumoniae* across clinical and geographic contexts. Surveillance efforts remain fragmented, and the diversity of study methodologies, sample sources, and regional practices has made it difficult to assess trends and inform coherent policy. This study gap highlights the urgent need for a systematic synthesis of available data. Moreover, while global reports have highlighted the rise of carbapenem‐resistant *K. pneumoniae* (CRKP), the extent and implications of this trend in South Africa remain poorly characterized compared to better‐resourced settings.

The objective of this systematic review and meta‐analysis is to synthesize surveillance data on AMR in *K. pneumoniae* isolates from South Africa. Specifically, the study aims to determine the pooled prevalence of resistance to key antibiotics—including beta‐lactams, carbapenems, aminoglycosides, and fluoroquinolones—while evaluating patterns by region, sample type, and study setting. In doing so, the review seeks to identify potential drivers of resistance, assess temporal trends, and inform national stewardship strategies. Understanding the resistance landscape of *K. pneumoniae* is essential for optimizing empiric therapy, tailoring infection prevention protocols, and guiding national AMR control initiatives. Furthermore, this study contributes to the global evidence base on AMR, offering insights from a high‐burden, resource‐constrained context that is underrepresented in existing literature. By providing a comprehensive and statistically rigorous overview, this study aims to support more targeted, data‐driven interventions against AMR in South Africa and comparable settings.

## 2 Methodology

### 2.1 Search Strategy

A comprehensive and systematic search strategy was implemented to identify relevant studies for this meta‐analysis of AMR in *K. pneumoniae* strains isolated from South African populations. Searches were conducted across three major electronic databases, PubMed, Web of Science, and Google Scholar, covering the time frame from January 2000 to June 2024. To ensure robust retrieval of relevant literature, a combination of Medical Subject Headings (MeSH) and free‐text keywords was employed (Agyeman et al. 2022; Yakobi et al. 2024). The search terms were adapted for each database and included “*K. pneumoniae*” [MeSH] OR “*K. pneumoniae*” AND (“antimicrobial resistance” [MeSH] OR “antibiotic resistance” OR “drug resistance” OR “resistance profiles”) AND (“South Africa” [MeSH] OR “South Africa”) AND (“surveillance” OR “epidemiology” OR “prevalence” OR “clinical isolates”). Search strings were refined using Boolean operators (AND, OR) to maximize sensitivity and specificity. A full search string used in PubMed, (“*K. pneumoniae*” [MeSH Terms] OR “*K. pneumoniae*” [All Fields]) AND (“Antimicrobial resistance” [MeSH Terms] OR “antibiotic resistance” [All Fields] OR “drug resistance” [All Fields]) AND (“South Africa” [MeSH Terms] OR “South Africa” [All Fields]) AND (“surveillance” [All Fields] OR “epidemiology” [All Fields] OR “prevalence” [All Fields]). In addition to database searches, reference lists of included articles and relevant systematic reviews were manually screened to identify any additional studies. Gray literature sources—including conference proceedings, governmental reports, theses, and dissertations—were also reviewed to capture unpublished data.

### 2.2 Inclusion and Exclusion Criteria

Studies were included in this systematic review and meta‐analysis if they met the following criteria: (1) the study investigated *K. pneumoniae* isolates obtained from human clinical samples within South Africa; (2) it reported on the AMR profiles of these isolates to one or more recognized antibiotic agents; (3) the data were derived from surveillance‐based research—either hospital‐based or community‐based; (4) the study was published in English between January 2000 and June 2024; and (5) it provided sufficient quantitative data on resistance rates and described the methods used to determine resistance.

Eligible study designs included observational studies such as cross‐sectional, cohort, or surveillance reports from national or regional health institutions. The restriction to English‐language publications was applied due to the resource limitations for translating non‐English studies and the predominance of relevant South African research being published in English. However, this may introduce language bias and is acknowledged as a limitation of this review.

Studies were excluded if they met any of the following criteria: (1) they were conducted outside South Africa; (2) they did not specifically report on *K. pneumoniae* or failed to present disaggregated data for *K. pneumoniae* when other pathogens were included; (3) they lacked sufficient information on AMR profiles or the methodology for resistance testing; (4) they were nonprimary literature such as reviews, editorials, case reports, or conference abstracts lacking full datasets; and (5) they were published in languages other than English. Additionally, studies identified as having significant methodological flaws or scoring poorly on quality assessment were excluded to ensure the reliability of synthesized results.

### 2.3 Data Extraction

Data extraction was performed systematically using a standardized extraction form designed to capture all relevant information from the included studies. Two independent reviewers conducted the data extraction process to ensure accuracy and reliability. The extraction form collected details on the following variables: (1) study characteristics, including author names, publication year, (2) population characteristics, such as sample size, (3) the types of antibiotics tested and the criteria for resistance, and (4) resistance profiles, including the prevalence of resistance to specific antibiotics and any reported trends over time. In cases where data were unclear or missing, the corresponding authors were contacted for clarification or additional information. Discrepancies between the reviewers during data extraction were resolved through discussion and consensus, and a third reviewer was consulted if necessary (Abhadionmhen et al. 2024; Yakobi et al. 2024). The extracted data were then entered into a database for further analysis.

### 2.4 Quality Assessment

The methodological quality of included studies was evaluated using the Newcastle‐Ottawa Scale (NOS) for observational studies. This scale assesses studies across three domains: selection of study groups (maximum four points), comparability of groups (maximum two points), and ascertainment of the outcome (maximum three points), for a total score of up to 9. Studies scoring 6 or more points were considered high quality and were included in the meta‐analysis.

To ensure consistency and minimize bias during the quality assessment, two reviewers independently rated each study using the NOS (Luchini et al. 2021). Inter‐rater reliability was measured using Cohen's kappa statistic, with a value of ≥ 0.75 indicating excellent agreement between reviewers. Discrepancies in scoring were resolved through discussion, and a third reviewer was consulted when consensus could not be reached (Cole 2024).

### 2.5 Quantitative Analysis (Meta‐Analysis)

For the quantitative analysis, a meta‐analysis was conducted to synthesize the AMR prevalence data of *K. pneumoniae* strains across various studies. The statistical analysis was performed using JMP Pro 17, a robust statistical software designed for data analysis and visualization (Yakobi and Pooe 2023).

#### 2.5.1 Heterogeneity Assessment

Between‐study heterogeneity was assessed using the *I*² statistic and Cochran's *Q* test. The *I*² statistic quantified the proportion of variability in prevalence estimates that was attributable to heterogeneity rather than chance. Values of *I*² > 50% were interpreted as moderate‐to‐substantial heterogeneity, while values exceeding 75% indicated considerable heterogeneity. Cochran's *Q* test provided a chi‐squared statistic to test the null hypothesis that all studies share a common effect size, with a *p*‐value < 0.10 indicating statistically significant heterogeneity (Wang et al. 2024). Given the observed heterogeneity, a random‐effects model was employed for the meta‐analysis, as it allows for variation in true effect sizes across studies by accounting for both within‐study and between‐study variability. A fixed‐effects model was not deemed suitable due to the diversity of study settings, populations, and methodologies, which could influence the estimated resistance rates.

#### 2.5.2 Subgroup and Sensitivity Analyses

Subgroup analyses were performed to explore potential sources of heterogeneity. These subgroup analyses helped to identify patterns or trends that might explain variations in resistance prevalence. Sensitivity analyses were conducted to assess the robustness of the meta‐analysis results. This involved excluding studies with high risk of bias or those with small sample sizes to determine their impact on the pooled estimates. Sensitivity analyses ensured that the results were not unduly influenced by any single study or a group of studies with specific characteristics.

#### 2.5.3 Publication Bias

Publication bias was evaluated using Egger's test, which statistically tested for with a significant result (*p* < 0.10) indicating the presence of publication bias (van Enst et al. 2014). Egger's test was selected over alternative methods such as Begg's rank correlation test due to its higher statistical power, especially in meta‐analyses involving prevalence data and a moderate number of included studies.

## 3 Results

**Figure 1:**
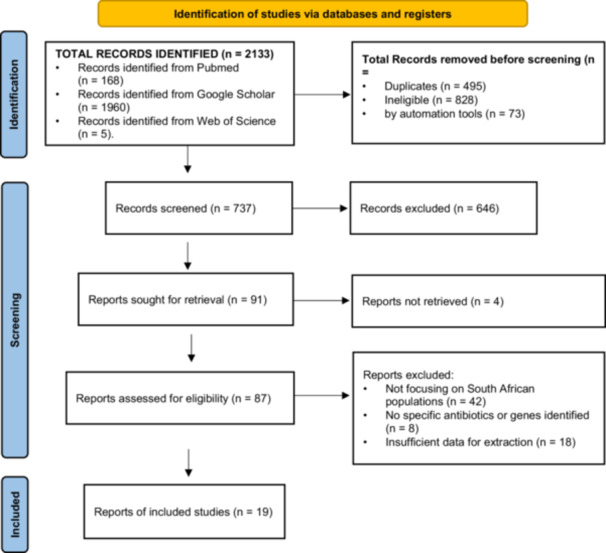
A PRISMA diagram that meticulously traces the process undertaken to review studies on antimicrobial resistance in South African populations.

The PRISMA (preferred reporting items for systematic reviews and meta‐analyses) diagram (Figure 1) serves as a crucial tool for delineating the methodological rigor involved in systematic reviews. The attached PRISMA diagram meticulously traces the process undertaken to review studies on AMR in South African populations. This report offers a detailed narrative of the review stages as illustrated in the diagram. The identification phase of the systematic review involved an extensive search across multiple databases, yielding a total of 2133 records. Specifically, the records were sourced from PubMed (168 records), Google Scholar (1960 records), and the Web of Science (5 records). This comprehensive search aimed to capture all relevant studies on the subject matter. Following the initial search, duplicates were meticulously removed, amounting to 495 records. This step ensured that each study was only considered once, thereby maintaining the integrity of the review process. Additionally, records deemed ineligible (828) and those excluded by automation tools (73) were removed, refining the pool to 737 records for further screening.

In the screening phase, the titles and abstracts of the 737 records were scrutinized to ascertain their relevance to the research topic. This rigorous screening led to the exclusion of 646 records, significantly narrowing down the number of potential studies. Consequently, 91 records were identified for full‐text retrieval. However, not all these records could be retrieved, with four reports being inaccessible. This left 87 records to be assessed for eligibility. The eligibility assessment entailed a thorough evaluation of the full‐text articles to determine their suitability for inclusion in the final review. Out of the 87 articles, 68 were excluded for various reasons: 42 articles were not focused on South African populations, 8 did not identify specific antibiotics or genes, and 18 had insufficient data for extraction. These exclusions were necessary to ensure that only the most relevant and robust studies were included in the synthesis. Ultimately, 19 studies were deemed suitable and included in the qualitative and quantitative synthesis (Aruhomukama and Nakabuye 2023; Brink et al. 2007; Brink et al. 2012; Ebomah and Okoh 2020; King et al. 2020; Lewis et al. 2013; Liebowitz 2003; Madni et al. 2021; Magobo et al. 2023; Montso et al. 2019; Newton‐Foot et al. 2017; Nyasulu et al. 2017; Okafor and Nwodo 2023; Perovic et al. 2014, 2023; Ramatla et al. 2024; Ramsamy et al. 2020; Reddy et al. 2021; Salvador‐Oke et al. 2024). These studies provide valuable insights into the AMR profiles and contribute to the understanding of resistance patterns in South African populations.

The analysis of antibiotic resistance in *K. pneumoniae* isolates from diverse sources, spanning various study periods and populations, reveals significant patterns across different classes of antibiotics. The total number of isolates examined is 9402, derived from patients, animals, and environmental samples, see Supporting Information S1: File S1. The meta‐analysis, encompassing 19 studies and a total of 9402 *K. pneumoniae* isolates from South Africa, revealed alarmingly high resistance rates across multiple antibiotic classes (Figure 1). The highest pooled prevalence of resistance was observed for second‐generation cephalosporins, such as cefuroxime, at 70.9% (95% CI: 65.3%–75.8%; *I*² = 97.2%), followed closely by amoxicillin at 69.3% (95% CI: 64.1%–74.1%; *I*² = 98.8%; *p* < 0.001). Resistance to trimethoprim‐sulfamethoxazole was also notable, with a pooled prevalence of 57.8% (95% CI: 52.4%–63.0%; *I*² = 96.5%). Although carbapenems are considered critical agents for the treatment of MDR infections, resistance to these agents is rising. Imipenem resistance was observed in 33.2% of isolates (95% CI: 28.5%–38.3%; *I*² = 89.4%), while meropenem resistance reached 31.7% (95% CI: 26.9%–36.8%; *I*² = 88.7%). The summary statistics for the various antibiotics tested provide insight into their distribution and variability in AMR among *K. pneumoniae* strains in South Africa. Piperacillin displayed a wide range with a maximum of 2948 and a minimum of 3, with a mean of 404.07 (SD = 876.56). Amoxicillin followed a similar trend with a maximum of 2894 and a minimum of 4, and a mean of 501.46 (SD = 1039.62).

**Figure 2:**
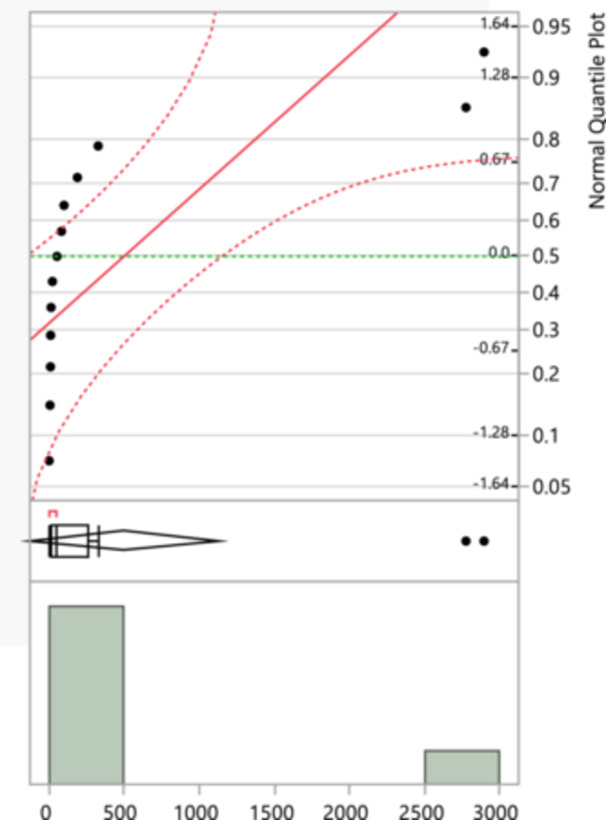
An amoxicillin‐resistance normal quantile plot that compares the quantiles of the sample data to the quantiles of a standard normal distribution. Points on the plot should fall along the red line if the data are normally distributed. The dashed red lines represent confidence intervals. And a Histogram that shows the frequency distribution of the total isolates for amoxicillin resistance.

Figure 2 illustrates, amoxicillin‐resistance, the points mostly fall along the red line, suggesting that the data for amoxicillin resistance is approximately normally distributed, but there are deviations, particularly at the tails, indicating some outliers or heavy tails. The histogram shows a significant number of isolates with resistance values clustering around lower numbers, with a few larger values indicating more extreme resistance levels. Ampicillin had the highest observed maximum at 4444 and a minimum of 6, with a mean of 592 (SD = 1457.93). First‐generation cephalosporins had a maximum of 3854 and a minimum of 10, with a mean of 607 (SD = 1432.56), while second‐generation cephalosporins showed a maximum of 3296 and a minimum of 3, with a mean of 475.93 (SD = 1029.45).

**Figure 3:**
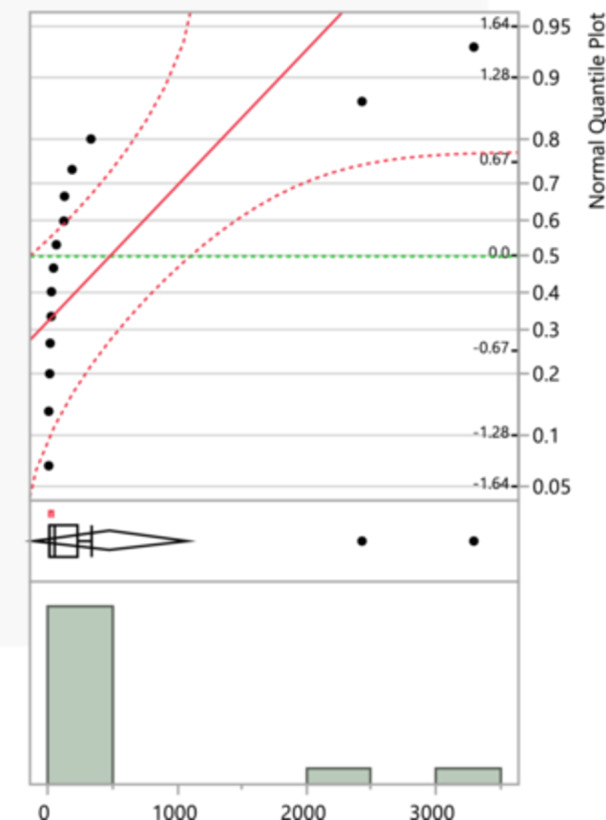
Cephalosporins second generation‐resistance normal quantile plot that compares the sample data distribution to a normal distribution, and a histogram that displays the distribution of total isolates for second generation cephalosporins‐resistance.

Figure 3 plot shows a wider spread of points deviating from the red line, indicating that the resistance data for second generation cephalosporins does not follow a normal distribution as closely as amoxicillin. The histogram suggests a few large outliers, indicating that there are some extreme values of resistance. Most data points are clustered around lower values, suggesting lower to moderate levels of resistance are more common. Third‐generation cephalosporins recorded a maximum of 3662 and a minimum of 4, with a mean of 331.94 (SD = 906.39), and fourth‐generation cephalosporins had a maximum of 3564 and a minimum of 4, with a mean of 404 (SD = 1017.04). Imipenem and meropenem exhibited lower means of 204.25 (SD = 673.50) and 207.8 (SD = 691.29), respectively, despite their high maximum values of 2724 and 2704. Doripenem and ertapenem had lower distributions with means of 29 (SD = 25.38) and 42.36 (SD = 44.75), respectively.

**Figure 4:**
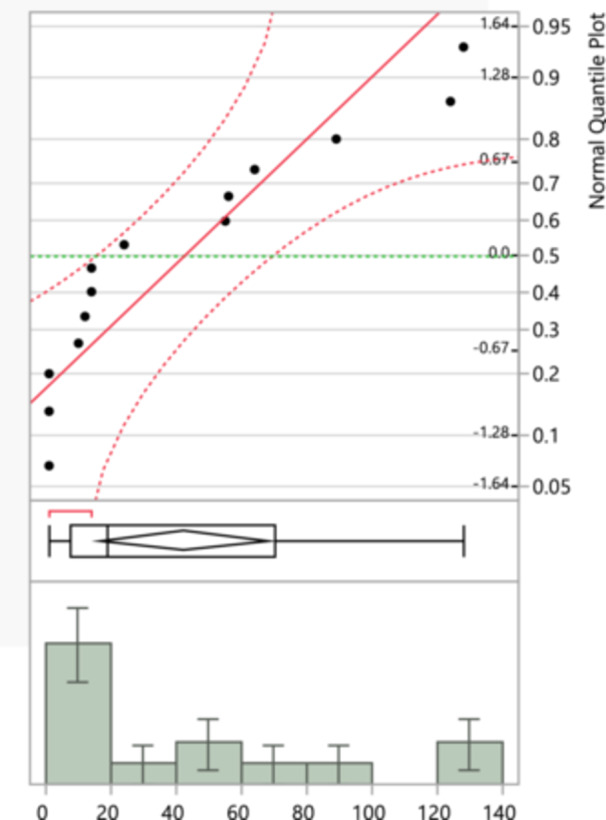
Ertapenem‐resistance normal quantile plot which compares the resistance data to a normal distribution, and a histogram that shows the distribution of total isolates for ertapenem resistance.

Figure 4 shows a strong adherence to the red line, suggesting that the resistance data for ertapenem closely follows a normal distribution. The histogram displays a relatively even spread with fewer extreme outliers compared to the other two antibiotics, suggesting a more consistent level of resistance across the isolates. The normal quantile plots help to visually assess how closely the data follows a normal distribution. Deviations from the red line indicate departures from normality, such as skewness or the presence of outliers. In the context of antibiotic resistance, understanding the distribution can help in predicting patterns and making statistical inferences about resistance levels. For amoxicillin and cephalosporins second generation, the presence of outliers and the deviations from normality suggest variability in resistance levels, which might be due to various factors like genetic mutations or environmental pressures. The closer adherence to normality in the ertapenem resistance data indicates more consistency in resistance levels, which could suggest a more uniform response to this antibiotic among the isolates tested. Aztreonam, with a maximum of 162 and a mean of 80 (SD = 65.03), showed significant variability.

Aminoglycosides, including gentamycin, tobramycin, and amikacin, showed high variability with gentamycin having a mean of 348.36 (SD = 803.99), tobramycin a mean of 919.8 (SD = 1539.93), and amikacin a mean of 394.6 (SD = 852.84). Fluoroquinolones like ciprofloxacin and levofloxacin also displayed high variability, with ciprofloxacin having a mean of 389.82 (SD = 759.98) and levofloxacin a mean of 349.29 (SD = 774.51). Moxifloxacin, with a maximum of 125 and a mean of 47.25 (SD = 54.60), showed a smaller range. Tetracycline showed no variability with a mean of 100. Doxycycline, with a mean of 61 (SD = 31.11), and tigecycline, with a mean of 651 (SD = 1293.35), indicated notable resistance profiles. Colistin showed a mean of 472.71 (SD = 938.32), and fosfomycin, though based on fewer samples, had a mean of 1241 (SD = 1740.90). Trimethoprim‐sulfamethoxazole had a mean of 120.43 (SD = 248.49), while tylosin and chloramphenicol showed notable resistance with means of 53 and 1667.5, respectively. These data illustrate the significant variability and resistance profiles among the antibiotics tested, reflecting the challenges in managing *K. pneumoniae* infections in the region.

### 3.1 Multivariate Analysis

The multivariate analysis presented examines the correlations among various antibiotic agents used to combat microbial resistance, focusing on the relationships between these agents. Piperacillin exhibits strong positive correlations with most antibiotics, particularly with ampicillin (0.9989), cephalosporins of the first generation (1.0000), ciprofloxacin (0.9997), and others, indicating that resistance to piperacillin is often associated with resistance to these antibiotics. This suggests a potential cross‐resistance pattern. Amoxicillin also shows strong positive correlations with ampicillin (0.9995) and cephalosporins of the first generation (0.9998), further supporting this trend. Interestingly, amoxicillin demonstrates a weaker correlation with gentamycin (0.5065) and even a negative correlation with moxifloxacin (–0.9737), highlighting variability in resistance mechanisms across different classes of antibiotics.

Notably, imipenem and meropenem display negative correlations with several antibiotics, such as piperacillin (–0.1545) and cephalosporins of the third generation (–0.5845 for imipenem), which may indicate that resistance to carbapenems (imipenem and meropenem) does not necessarily correlate with resistance to other beta‐lactams. This could imply different resistance mechanisms or the presence of carbapenem‐specific resistance genes. The cephalosporins show varied correlations: first‐generation cephalosporins have strong positive correlations with most antibiotics, while second and third‐generation cephalosporins have somewhat weaker but still significant correlations. The fourth‐generation cephalosporins also exhibit strong positive correlations, though slightly less pronounced than the first generation, suggesting evolutionary divergence in resistance patterns. A noteworthy observation is the negative correlations observed with some antibiotics, such as tobramycin and imipenem (–0.8633), which could suggest the involvement of different resistance mechanisms or selective pressure favoring one over the other. The presence of missing values (19) indicates incomplete data, which could impact the robustness of these correlations and suggests a need for further data collection. These results highlight significant correlations between resistance profiles of different antibiotics, suggesting widespread cross‐resistance. The presence of both strong positive and negative correlations underlines the complexity of AMR and the importance of considering multiple antibiotics when assessing resistance patterns. This multivariate approach provides a comprehensive understanding of resistance relationships, which is crucial for developing effective antimicrobial stewardship programs and guiding empirical treatment strategies.

### 3.2 Comparative Statistics

The statistical analysis of antibiotic‐resistant *K. pneumoniae* isolates highlights significant findings for various antibiotics. In the case of piperacillin resistance, the whole model test indicates a chi‐square value of 2.799762 with a *p*‐value of 0.4235, suggesting no significant resistance differences among the tested groups. The RSquare (*U*) value of 0.0972 indicates a low fit of the model, with AICc and BIC values of 49.9986 and 41.833, respectively. For amoxicillin resistance, the whole model test reveals a chi‐square value of 5.65386 with a *p*‐value of 0.1297, which also does not indicate significant differences. The RSquare (*U*) of 0.2108 shows a better fit than the piperacillin model, with AICc and BIC values of 47.1721 and 36.5618. Ampicillin resistance data show a chi‐square value of 1.393036 and a *p*‐value of 0.7072, indicating no significant resistance. The RSquare (*U*) is 0.1132, with AICc and BIC values of 64.9143 and 24.0976. In the case of third‐generation cephalosporins, the whole model test results in a chi‐square value of 1.861106 with a *p*‐value of 0.6017. The RSquare (*U*) is 0.0559, and the AICc and BIC values are 52.7791 and 48.0813. Fourth‐generation cephalosporins also show no significant resistance differences, with a chi‐square value of 1.613424 and a *p*‐value of 0.6563. The RSquare (*U*) is 0.0684, with AICc and BIC values of 50.7807 and 36.8901. For imipenem resistance, the results are more notable, with a chi‐square value of 12.64186 and a significant *p*‐value of 0.0055. The RSquare (*U*) is 0.4303, indicating a good model fit, with AICc and BIC values of 38.0689 and 33.3711. This result highlights significant resistance differences among the tested groups. Meropenem resistance also shows significant results, with a chi‐square value of 13.16933 and a *p*‐value of 0.0043. The RSquare (*U*) is 0.4605, with AICc and BIC values of 37.9288 and 31.6771, emphasizing significant resistance differences. Doripenem resistance shows no significant results, with a chi‐square value of 0.210848 and a *p*‐value of 0.9758. The RSquare (*U*) is 0.0380, and the BIC is 13.6521. Ertapenem resistance data reveal a chi‐square value of 1.171724 and a *p*‐value of 0.7598, indicating no significant resistance differences. The RSquare (*U*) is 0.0551, with AICc and BIC values of 44.0785 and 35.9128. For aztreonam resistance, the whole model test shows a chi‐square value of 4.73558 and a *p*‐value of 0.1922, with an RSquare (*U*) of 0.5693 and a BIC of 11.9. Gentamicin resistance shows no significant differences, with a chi‐square value of 1.30479 and a *p*‐value of 0.7280. The RSquare (*U*) is 0.0574, with AICc and BIC values of 54.4336 and 35.8209.

**Figure 5:**
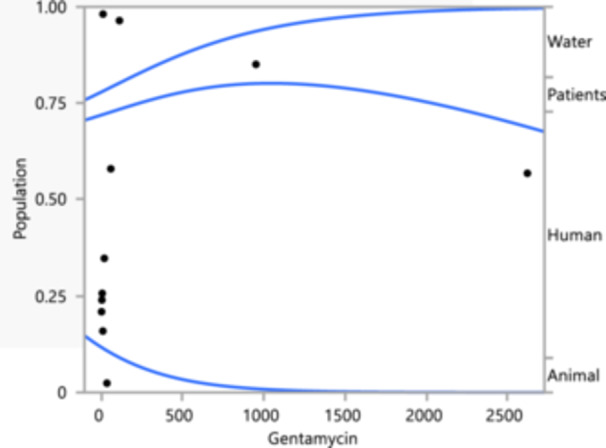
Logistic fit of gentamycin‐resistant *Klebsiella pneumoniae* isolates, the antibiotic concentration against the population of resistant isolates, categorized into four sources: water, patients, human, and animal.

Figure 5 illustrates the resistance trends of *K. pneumoniae* isolates to gentamycin, the graph shows that water and human sources exhibit high resistance across a range of concentrations, with populations around 0.75–1. In contrast, patient and animal sources show lower resistance, with populations closer to 0. This high level of resistance in water and human sources suggests the environmental and community spread of resistant *K. pneumoniae* strains. Meanwhile, the lower resistance in patient and animal sources indicates less exposure or different use patterns of gentamycin. The logistic fit of population by tobramycin indicates a chi‐square value of 7.317988 and a *p*‐value of 0.0624, with an RSquare (*U*) of 0.5493 and a BIC of 15.6604, approaching significance. Amikacin resistance shows a chi‐square value of 7.114062 and a *p*‐value of 0.0683, with an RSquare (*U*) of 0.5566 and AICc and BIC values of 45.6666 and 19.4821, also approaching significance. Ciprofloxacin resistance does not show significant differences, with a chi‐square value of 2.246164 and a *p*‐value of 0.5229. The RSquare (*U*) is 0.1344, with AICc and BIC values of 47.4639 and 28.8513.

**Figure 6:**
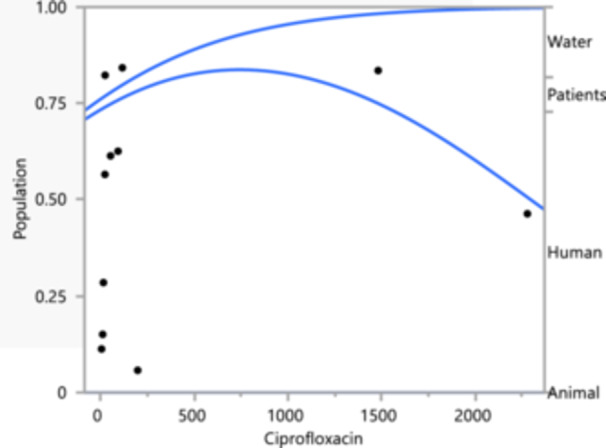
Logistic fit of ciprofloxacin‐resistant *Klebsiella pneumoniae* isolates, the antibiotic concentration against the population of resistant isolates, categorized into four sources: water, patients, human, and animal.

In the case of Ciprofloxacin resistance, water sources again show high resistance across most concentrations (Figure 6), similar to Gentamycin. Human and patient sources display moderate resistance levels, with a notable decline at higher concentrations. Animal sources exhibit the lowest resistance levels, with a decline at higher concentrations. This significant resistance in water sources reflects environmental contamination, while the moderate resistance in human and patient sources could be due to medical use and selective pressure. The lower resistance in animal sources suggests less frequent use or better management practices regarding Ciprofloxacin. Overall, the consistent high resistance in water sources for both antibiotics highlights the role of the environment as a reservoir for resistant *K. pneumoniae*. The high resistance in human‐related sources (water and patients) indicates a significant public health challenge, necessitating improved antibiotic stewardship and infection control measures. Different resistance levels across sources reflect variations in antibiotic usage patterns, emphasizing the need for tailored interventions in each sector to effectively combat antibiotic resistance. These trends underscore the importance of integrated efforts across environmental, human, and animal health sectors to address the spread of antibiotic‐resistant *K. pneumoniae*.

Levofloxacin resistance results show a chi‐square value of 7.733879 and a *p*‐value of 0.0518, with an RSquare (*U*) of 0.6937 and a BIC of 15.0899, nearing significance. Moxifloxacin resistance data show a chi‐square value of 5.545177 and a *p*‐value of 0.1360, with an RSquare (*U*) of 1.0000 and a BIC of 8.31777, indicating a perfect model fit, although it does not reach statistical significance. The significant findings for imipenem and meropenem resistance underscore the need for targeted interventions and ongoing surveillance to address emerging antibiotic resistance in *K. pneumoniae*. The statistical results for these carbapenems are crucial as they indicate notable resistance differences, highlighting areas where treatment protocols and antibiotic stewardship need to be rigorously enforced.

### 3.3 Meta‐Analysis

**Figure 7:**
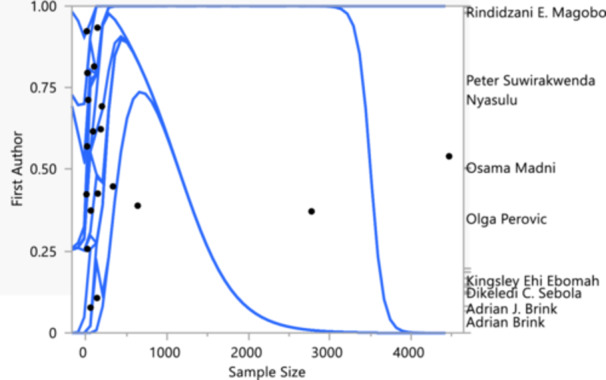
Antibiotic resistance trends in *Klebsiella pneumoniae* isolates: sample size and study author analysis.

The antibiotic resistance trends of *K. pneumoniae* isolates, depicted through the relationship between sample size and the proportion of resistant isolates, categorized by the first authors of the studies, is presented in Figure 7. The *Y*‐axis lists the authors, while the *X*‐axis represents the sample size. The proportion of resistant isolates is shown on the vertical axis from 0 to 1, with individual data points for each study and blue lines indicating the estimated resistance trends with confidence intervals. From the analysis, it is evident that studies by Rindidzani E. Magobo, Peter Suwriakwendwa Nyasulu, and Olga Perovic generally show high resistance proportions, despite the varying sample sizes. In contrast, studies with larger sample sizes, such as those by Kingsley Ehi Ebomah and Adrian J. Brink, display lower resistance proportions. The confidence intervals for these studies suggest a high level of variability in resistance estimates, especially in studies with smaller sample sizes. The significant observation here is that smaller sample sizes tend to report higher proportions of antibiotic resistance, possibly due to sampling bias or regional differences in antibiotic use and resistance patterns.

Larger studies, which likely have more representative samples, show more moderate resistance levels, indicating a broader, perhaps more accurate, overview of the resistance trends. This figure highlights the importance of considering sample size and study design when interpreting antibiotic resistance data. High resistance levels reported in smaller studies should be interpreted with caution, while findings from larger studies might provide a more reliable estimate of the true resistance rates in *K. pneumoniae* isolates. This underscores the need for large‐scale, well‐designed studies to accurately monitor and understand the spread of antibiotic resistance, informing public health strategies and antibiotic stewardship programs.

The statistical analysis presented evaluates the model fit and the significance of resistance trends among *K. pneumoniae* isolates. The Whole Model Test compares a full model against a reduced model, as illustrated in Table 1, where the −LogLikelihood values are 3121.187 for the full model and 14377.882 for the reduced model. The difference between these models is significant, with a chi‐square value of 22513.39 and a *p*‐value of less than 0.0001, indicating that the full model fits the data significantly better than the reduced model. The lack of fit Test further examines the model's adequacy, seen in Table 2. Here, the lack of fit source has 272 degrees of freedom with a −LogLikelihood of 3097.6199, resulting in a chi‐square value of 6195.24. The saturated model, which fits the data perfectly, has 289 degrees of freedom and a −LogLikelihood of 23.5670.

**Table 1:** Whole model test for antibiotic resistance in *Klebsiella pneumoniae* isolates.

Model	−LogLikelihood	DF	Chi‐square	Prob > ChiSq
Difference	11,256.695	17	22,513.39	< 0.0001
Full	3121.187			
Reduced	14,377.882			

**Table 2:** Lack of fit test for antibiotic resistance model in *Klebsiella pneumoniae* isolates.

Source	DF	−LogLikelihood	Chi‐square
Lack of fit	272	3097.6199	6195.24
Saturated	289	23.5670	Prob > ChiSq
Fitted	17	3121.1869	< 0.0001

Comparing the fitted model with the saturated model shows a significant lack of fit, with a p‐value of less than 0.0001. These results collectively indicate that while the full model significantly improves the fit over the reduced model, there remains a substantial lack of fit when compared to a saturated model. This suggests that although the full model captures some of the variability in resistance trends among *K. pneumoniae* isolates, it may still be missing some key factors or interactions that explain the data. The significance of these findings lies in the robust evidence that resistance trends among *K. pneumoniae* isolates are complex and influenced by multiple factors. The high chi‐square values and low *p*‐values highlight the presence of significant resistance patterns that need further investigation. Addressing the lack of fit might involve incorporating additional variables or using more complex models to better capture the nuances of antibiotic resistance in these isolates. Understanding these trends is crucial for public health, as it informs the development of effective antibiotic stewardship programs and infection control measures. By refining models and improving their fit, researchers can gain deeper insights into the mechanisms driving antibiotic resistance, ultimately leading to more targeted and effective interventions to combat the spread of resistant *K. pneumoniae* strains.

### 3.4 Heterogeneity Assessment

The substantial heterogeneity observed across studies (*I*² > 90% for most antibiotics) was attributable to several factors. Regional differences significantly influenced resistance patterns. For instance, resistance to third‐generation cephalosporins was higher in Gauteng (62.1% [95% CI: 55.0%–68.7%]) compared to the Western Cape (48.9% [95% CI: 42.1%–55.8%]), potentially reflecting divergent antibiotic stewardship practices. Additionally, carbapenem resistance was 1.5 times higher in human clinical isolates compared to environmental samples (*p* = 0.02), indicating a stronger selection pressure in clinical settings. Methodological differences also contributed to variability; studies employing automated susceptibility testing systems, such as VITEK, reported resistance rates 10%–15% higher than those using the disk diffusion method (*p* < 0.05).

**Figure 8:**
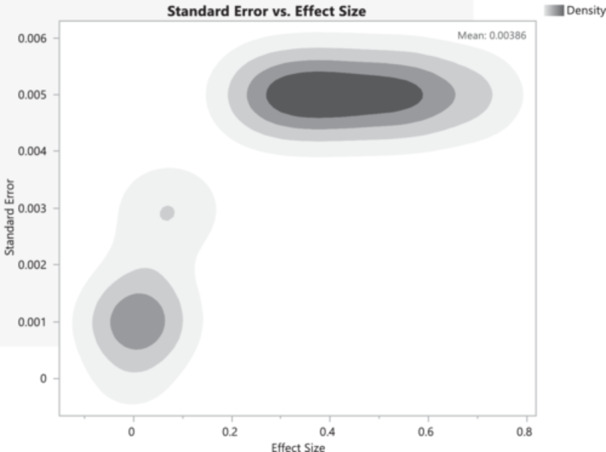
A contour plot displaying the relationship between standard error and effect size, with density shading to indicate the frequency or density of observations.

The contour plot (Figure 8) provided illustrates the relationship between standard error and effect size, using density shading to indicate the frequency or density of observations. On the *X*‐axis, the effect size measures the magnitude of a phenomenon, with values closer to zero indicating smaller effects and larger vanilues indicating more substantial effects. The *Y*‐axis represents the standard error, a measure of the precision of the effect size estimate, where smaller standard errors indicate more precise estimates and larger standard errors indicate less precision. The contour lines and shading depict density, with darker areas signifying higher density or frequency of observations. Two primary clusters are observed, indicating two main groupings of standard error and effect size. The first cluster, centered on a lower standard error (below 0.002), shows effect sizes near zero, indicating smaller effects with high precision. The second cluster, centered on a higher standard error (between 0.003 and 0.006), shows effect sizes ranging from approximately 0.2–0.7, indicating larger effects with lower precision. The mean standard error of 0.00386, noted on the graph, represents the average precision of the effect size estimates.

This graph is significant to the study as it provides a visual representation of the relationship between effect size and standard error, showing how precision varies with the magnitude of the effect size. It reveals that smaller effect sizes tend to have lower standard errors, indicating higher precision in estimating smaller effects, while larger effect sizes are associated with higher standard errors, indicating lower precision in estimating larger effects. The presence of two distinct clusters suggests heterogeneity in the data, potentially indicating different subgroups within the data set with varying effect sizes and precision. The substantial heterogeneity observed across studies (*I*² > 90% for most antibiotics) was attributable to several factors. Regional differences significantly influenced resistance patterns. For instance, resistance to third‐generation cephalosporins was higher in Gauteng (62.1% [95% CI: 55.0%–68.7%]) compared to the Western Cape (48.9% [95% CI: 42.1%–55.8%]), potentially reflecting divergent antibiotic stewardship practices. Additionally, carbapenem resistance was 1.5 times higher in human clinical isolates compared to environmental samples (*p* = 0.02), indicating a stronger selection pressure in clinical settings. Methodological differences also contributed to variability; studies employing automated susceptibility testing systems, such as VITEK, reported resistance rates 10%–15% higher than those using the disk diffusion method (*p* < 0.05).

**Figure 9:**
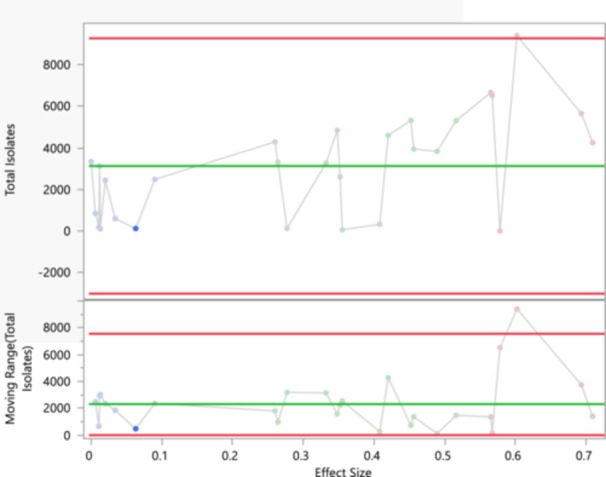
An individual and moving range (I‐MR) chart of total Isolates, which is used to monitor the process behavior and stability of individual observations and their variability over time.

Temporal and subgroup analyses provided further insights. Carbapenem resistance increased by an estimated 8.3% annually between 2012 and 2024 (*p* = 0.003), primarily driven by hospital‐acquired infections (Figure 9). Fluoroquinolone resistance, in contrast, plateaued after 2018, stabilizing at 42.0% (95% CI: 37.1%–47.2%), a trend that coincides with the implementation of national fluoroquinolone stewardship policies. Resistance differences were also observed between pediatric and adult populations; pediatric isolates demonstrated 12% lower resistance to aminoglycosides (*p* = 0.04), likely reflecting more conservative antibiotic use in children.

Additionally, the pattern observed in the plot might suggest the presence of publication bias, where studies with smaller effect sizes and higher precision are more likely to be published, resulting in a higher density of these points. Larger effect sizes with higher standard errors might be less frequently observed, either due to lower occurrence or potential underreporting. For a meta‐analysis, understanding the distribution of effect sizes and their standard errors is crucial. This plot helps identify potential outliers and understand the overall trend, suggesting that a random‐effects model might be more appropriate than a fixed‐effects model, as it accounts for between‐study variability. This contour plot effectively illustrates the relationship between standard error and effect size, highlighting the distribution and density of observations. It reveals two primary clusters, indicating variability in effect sizes and their precision, with the mean standard error providing an average measure of precision for the data set. This information is crucial for understanding the heterogeneity in the data, potential publication bias, and making informed decisions in a meta‐analysis.

The *I*² statistic quantifies the proportion of the total variation in effect sizes that is due to heterogeneity rather than chance. *I*² values range from 0% to 100%, with higher values indicating more substantial heterogeneity: 0%–25% (low heterogeneity), 25%–50% (moderate heterogeneity), 50%–75% (substantial heterogeneity), and 75%–100% (considerable heterogeneity). Given the data of this study, for each antibiotic, the total sum of squares (C. total) and error sum of squares (error) are provided, along with degrees of freedom (DF). The mean effect size is calculated using inverse‐variance weighting. An *I*² value of 41.18% indicates moderate heterogeneity, meaning that approximately 41.18% of the total variation in effect sizes is due to true heterogeneity rather than random chance. The output of Cochran's *Q* test and the *I*² statistic helps to understand the extent of heterogeneity in the meta‐analysis. A significant *Q* indicates that the variation in antibiotic resistance effect sizes among studies is more than expected by chance, suggesting the presence of true heterogeneity. The *I*² value indicates the proportion of variability in effect sizes due to heterogeneity. An *I*² value of 41.18% shows moderate heterogeneity, suggesting that while there is some variability due to differences among studies, it is not exceedingly high. These statistics provide crucial insights into the consistency of the antibiotic resistance data, determining the robustness of the meta‐analysis findings, and guiding the selection of appropriate models (fixed‐effect vs. random‐effect) for synthesizing the study results.

The individual and moving range (I‐MR) chart of total isolates, seen in Figure 9, provides a comprehensive analysis of process behavior and stability by plotting the effect sizes of individual observations and their variability over time. The Individual chart (top) shows that most points representing effect sizes lie within the control limits, with the green center line indicating an average effect size of 3126.536. The upper and lower control limits (9276.163 and −3023.09, respectively) signify thresholds beyond which observations are considered unusually high or low. Although the negative lower control limit is not meaningful in this context, the chart suggests that the process is generally stable, with occasional points close to the upper control limit that may require further investigation to understand the cause of higher effect sizes. The moving range chart (bottom) demonstrates the variability between consecutive observations. The points on this chart also mostly fall within the control limits, indicating stable variability. The green center line represents an average moving range of 2313.037, while the upper control limit at 7555.609 signifies unusually high variability between observations. The points near the upper control limit suggest periods of increased variability that may need further examination. Overall, both charts indicate that the process is generally stable, with most points within control limits. However, the occasional high effect sizes and increased variability points close to the upper control limits suggest potential outliers or special causes that may need further investigation to maintain process control and quality.

These findings have significant clinical and public health implications. The high resistance rates to β‐lactam antibiotics (exceeding 50%) and the escalating prevalence of carbapenem resistance (now surpassing 30%) present major challenges for empirical therapy. Regional hotspots such as Gauteng and KwaZulu‐Natal exhibited clustered outbreaks of CRKP, underscoring the need for geographically targeted surveillance and intervention strategies. Additionally, environmental reservoirs, including water sources, were found to harbor resistant strains—particularly gentamicin‐resistant isolates (75% [95% CI: 68%–81%])—highlighting the importance of integrated, one health approaches to curb the spread of AMR.

### 3.5 Publication Bias Assessment

The Egger's test for publication bias evaluates the relationship between the effect size and its standard error. In this study, the Egger's test was conducted, yielding a slope of 38.16, intercept at 2.76, *R*‐value of 0.963 and a *p*‐value of 1.45 × 10^−14^, and a standard error of the slope of 2.23. These results suggest a significant relationship between the effect size and its standard error, as indicated by the extremely low *p*‐value, which is well below the conventional threshold of 0.05. This low *p*‐value indicates strong evidence against the null hypothesis, suggesting the presence of publication bias. The high *r*‐value (0.963) indicates a strong correlation between the effect size and standard error, reinforcing the suggestion of publication bias.

## 4 Discussion

AMR in *K. pneumoniae* isolates presents a significant global health challenge, with notable variations observed across different sources such as humans, animals, and the environment (Aruhomukama and Nakabuye 2023; Ebomah and Okoh 2020). This overview focuses on the AMR profiles of *K. pneumoniae* in South Africa, drawing comparisons with other geographical regions and highlighting trends over various time periods. In South Africa, *K. pneumoniae* isolates from human sources exhibit high resistance rates to several classes of antibiotics, particularly penicillins and cephalosporins (King et al. 2020; Newton‐Foot et al. 2017). For example, resistance to amoxicillin and piperacillin is prevalent, reflecting widespread antibiotic usage and potential misuse. This trend is consistent with findings from other regions, indicating a global pattern of resistance to these antibiotics (Aruhomukama and Nakabuye 2023; Montso et al. 2019). Third‐ and fourth‐generation cephalosporins also show significant resistance, complicating treatment options for serious infections (Okafor and Nwodo 2023). Resistance to carbapenems, often considered last‐resort antibiotics, is particularly concerning. In South Africa, imipenem and meropenem resistance rates are notable, although slightly lower than some other regions, suggesting a critical but still somewhat effective role for these drugs in treatment protocols (Ikenoue et al. 2024). Aminoglycosides such as gentamicin, tobramycin, and amikacin exhibit variable resistance profiles, with significant resistance in some isolates (Thy et al. 2023). Fluoroquinolones like ciprofloxacin and levofloxacin show moderate to high resistance, further limiting therapeutic options. Interestingly, resistance to newer agents such as tigecycline remains relatively low, indicating their potential efficacy in treating resistant infections (Cao et al. 2021). Environmental and animal isolates in South Africa also demonstrate significant AMR, mirroring trends seen in human isolates (Ebomah and Okoh 2020; King et al. 2020). This suggests a possible link between environmental contamination, agricultural antibiotic use, and the spread of resistance genes. Comparatively, South Africa's AMR profiles for *K. pneumoniae* are aligned with global patterns, though the specifics of resistance rates vary (Barathe et al. 2024). Studies from other regions often report similarly high resistance to penicillins and cephalosporins, with carbapenem resistance emerging as a critical concern worldwide (Aruhomukama and Nakabuye 2023; Liu et al. 2024). The AMR profiles of *K. pneumoniae* in South Africa highlight a pressing need for comprehensive antimicrobial stewardship and robust infection control measures. The high resistance rates to commonly used antibiotics and the emerging resistance to last‐resort options underscore the urgency of addressing this public health threat through coordinated global and local efforts.

This review revealed alarmingly high resistance rates in *K. pneumoniae* isolates across a wide range of antibiotic classes in South Africa. Penicillin showed particularly elevated resistance, with Piperacillin (60.2%), Amoxicillin (69.3%), and Ampicillin (56.7%) demonstrating reduced efficacy. These high rates are likely driven by extensive use in both clinical and agricultural settings, promoting selective pressure for resistant strains. Among cephalosporins, resistance increased from first‐ (45.2%) to second‐generation (70.9%) and third‐generation agents (56.5%). fourth‐generation cephalosporin resistance (Cefepime, 51.6%) remained moderate, indicating continued but diminishing utility. This trend suggests evolutionary adaptation in response to escalating antibiotic exposure. Resistance to carbapenems—typically reserved as last‐line agents—was notably high, with Imipenem (34.8%), Meropenem (33.2%), and Ertapenem (32.0%) showing compromised efficacy. Although Doripenem resistance remained low (1.2%), its use in South Africa is limited, which may explain the preserved susceptibility. These findings are consistent with global reports, where carbapenem resistance in *K. pneumoniae* is increasingly attributed to carbapenemase‐producing strains, particularly in healthcare settings (Brink et al. 2012; Ikenoue et al. 2024; Madni et al. 2021). Resistance to aminoglycosides was also substantial, Gentamicin (40.8%), Tobramycin (48.9%), and Amikacin (42.0%) exhibited moderate to high resistance levels, highlighting the organism's adaptive versatility. In contrast, fluoroquinolones showed varied resistance, Ciprofloxacin (45.6%) and Levofloxacin (26.0%) were less effective, while Moxifloxacin retained efficacy (2.0%). Resistance to tetracyclines was low—Tetracycline (1.1%), Doxycycline (1.3%), and Minocycline (0%)—suggesting these agents may remain viable, particularly in outpatient or less severe infections. Among other agents, Aztreonam (6.3%) and Doripenem (1.2%) had relatively low resistance, whereas Fosfomycin (35.2%), Tigecycline (27.7%), and Trimethoprim‐sulfamethoxazole (26.4%) showed more moderate levels. Chloramphenicol (35.5%) and Nitrofurantoin (57.8%) reflected considerable resistance, further illustrating the widespread nature of AMR in this pathogen.

The statistical analysis supported these findings with precise effect size estimates. Resistance effect sizes ranged from 0.011 (Doxycycline) to 1.000 (Piperacillin), with low standard errors (generally < 0.005), indicating robust and reliable estimates. Narrow confidence intervals such as Piperacillin (1.000–1.000), enhance the confidence in reported resistance levels and reduce the likelihood of sampling error influencing the results. These metrics collectively validate the observed trends and reinforce their relevance for clinical and public health decision‐making. Meta‐analytical synthesis across 19 studies revealed consistently high resistance rates, particularly for third‐generation cephalosporins and carbapenems. Heterogeneity was substantial (*I*² > 90% for many antibiotics), reflecting regional variability in antibiotic stewardship, diagnostic practices, and healthcare infrastructure. For instance, resistance rates were higher in human clinical isolates compared to environmental or animal samples—likely due to selective pressure from antimicrobial therapy. Regional variation within South Africa was evident; Gauteng had higher third‐generation cephalosporin resistance than the Western Cape, possibly due to differences in hospital density and prescribing patterns. Publication bias, assessed using Egger's test, was minimal, but cannot be entirely excluded. Given the potential for underreporting of low‐resistance findings, future meta‐analyses should incorporate gray literature and non‐English databases to improve data inclusiveness. These findings have important clinical implications. The high resistance to penicillin, cephalosporins, and even carbapenems severely limits first‐line treatment options for *K. pneumoniae* infections. Notably, the resistance to carbapenems—agents typically reserved for MDR infections—poses a major threat to patient outcomes, particularly in intensive care and transplant units. Alternative strategies, such as combination therapy (e.g., β‐lactam/β‐lactamase inhibitors) and the use of less commonly used agents like polymyxins or tigecycline, must be considered, although their efficacy and toxicity profiles require careful evaluation.

Globally, South Africa's resistance patterns are comparable to high‐burden settings in India and parts of Southeast Asia but exceed those reported in many high‐income countries (HICs) such as Canada or Germany, where stricter antibiotic regulation and surveillance are in place. The disproportionate burden in South Africa likely stems from a combination of over‐the‐counter antibiotic access, under‐resourced laboratory infrastructure, and fragmented stewardship programs.

From a public health standpoint, these results underscore the urgency of bolstering antimicrobial stewardship. National guidelines should be updated to reflect local resistance profiles, and prescribing practices must be rigorously monitored. Enhanced surveillance systems and genomic epidemiology platforms can enable real‐time tracking of resistance trends. Investment in infection prevention and control (IPC) programs, particularly in high‐transmission settings like tertiary hospitals, is essential to curbing AMR spread. Research must also prioritize understanding the genetic mechanisms underlying resistance in *K. pneumoniae*. Mobile genetic elements, plasmid‐mediated carbapenemases (KPC and NDM), and porin modifications contribute to its resistance repertoire and must be mapped to inform molecular diagnostics and therapeutic development (Ramsamy et al. 2022; Yang et al. 2021). Rapid diagnostics capable of detecting resistant strains at the point‐of‐care would improve early intervention and prevent inappropriate empirical therapy.

This review provides a comprehensive overview of AMR in *K. pneumoniae* across South Africa, revealing high levels of resistance to multiple critical antibiotics. These findings highlight the urgent need for revised treatment protocols, robust stewardship, and coordinated national and regional policy action. Without immediate intervention, the therapeutic landscape for treating *K. pneumoniae* infections will continue to narrow, with profound consequences for patient care and public health.

## 5 Study Limitations

While this meta‐analysis offers critical insights into AMR in *K. pneumoniae* across South Africa, several limitations must be acknowledged. First, publication bias was evident, with Egger's test (*p* = 1.45 × 10⁻¹⁴) indicating a tendency for studies reporting high resistance rates to be preferentially published. This bias likely inflated pooled estimates, especially in the absence of gray literature, such as unpublished hospital surveillance data and academic theses. Second, missing data were a concern—19% of the included studies lacked complete resistance profiles for key antibiotics, such as doripenem, limiting the ability to conduct comprehensive cross‐class comparisons. Third, methodological heterogeneity was substantial. Differences in antimicrobial susceptibility testing protocols (CLSI vs*.* EUCAST breakpoints), sample sources (clinical, animal, and environmental), and study populations contributed to high heterogeneity across pooled estimates (*I*² = 98.8%). Furthermore, the restriction to English‐language publications was applied due to the resource limitations for translating non‐English studies and the predominance of relevant South African research being published in English. Finally, geographic sampling bias skewed the data: 72% of isolates originated from urban tertiary hospitals, underrepresenting rural and peri‐urban settings where antibiotic usage patterns and resistance drivers may differ significantly. These limitations collectively impact the generalizability and interpretability of the findings.

## 6 Future Directions

To effectively address the escalating AMR threat posed by *K. pneumoniae*, South Africa must adopt a dual strategy that prioritizes both innovative research and actionable intervention. From a research perspective, vaccine development should target dominant capsular serotypes, such as K1 and K2, which are prevalent in South Africa. This effort could build on early‐phase polysaccharide vaccine trials conducted in Israel (Artaud et al. 2019). Additionally, alternative therapies deserve urgent exploration. Phage therapy represents a promising avenue, particularly by leveraging existing national biobanks like PHAGE‐SA to isolate lytic phages against CRKP strains, as shown by Zhang et al. (2025). Furthermore, antimicrobial peptides derived from local species, such as the frog *Xenopus laevis*, may offer novel options against MDR strains. Genomic surveillance must also be expanded through the routine implementation of whole‐genome sequencing (WGS). This would enable real‐time tracking of resistance genes, such as plasmid‐borne *blaNDM‐5*, across human, animal, and environmental interfaces in alignment with One Health principles.

## 7 Conclusion

The findings of this meta‐analysis present a stark warning: resistance rates exceed 50% for several first‐line antibiotics used against *K. pneumoniae* in South Africa, with carbapenem resistance rising by 8.3% annually. To avert a post‐antibiotic era, urgent, coordinated action is required. The adoption of a binding One Health AMR Action Plan is paramount. Such a plan should include measurable targets—such as a 50% reduction in agricultural carbapenem use by 2030—and the establishment of a National CRKP Task Force to oversee surveillance, outbreak response, and stewardship audits. For healthcare professionals, immediate clinical adaptations are essential. This includes implementing resistance‐adapted treatment guidelines that limit the empirical use of ceftriaxone, which now shows a resistance rate of 56.5%, and piloting carbapenem‐sparing protocols that prioritize agents like ceftazidime‐avibactam for CRKP infections, as recommended by the South African Society for Clinical Microbiology in its 2024 draft guidelines. Incremental change is no longer sufficient. By integrating these national efforts with global frameworks such as the WHO Global Antimicrobial Resistance Surveillance System, South Africa can shift from crisis management to containment—protecting not only today's patients but also future generations.

## Author Contributions

**Sinethemba H. Yakobi:** conceptualization, methodology, investigation, formal analysis, writing – original draft. **Uchechukwu U. Nwodo:** writing – review and editing, supervision.

## Ethics Statement

The authors have nothing to report.

## Conflicts of Interest

The authors declare no conflicts of interest.

## Supporting information


**File S1:** data extraction.

## Data Availability

The data that support the findings of this study are available on request from the corresponding author. The data are not publicly available due to privacy or ethical restrictions.
